# Human immunodeficiency virus therapeutics and pharmacogenomics

**DOI:** 10.4103/0971-6866.80354

**Published:** 2011-05

**Authors:** U. Shankarkumar, A. Shankarkumar, K. Ghosh

**Affiliations:** National Institute of Immunohaematology, 13^th^ floor, KEM Hospital, Parel, Mumbai, Maharashtra, India

## Abstract

Pharmacogenomics and pharmacogenetics are promising in development of a personalized treatment approach They are of paramount importance for basic immunology, for peptide based vaccine design (vaccinomics) drug monitoring in clinical setting and molecular pathophysiology of multifactorial diseases like cancer, tuberculosis, cardiac disorders, diabetes, asthma, HIV, etc

Pharmacogenomics is the branch of Pharmacology which deals with the influence of genetic variation on drug response in diseased person studied by correlating gene expression and/or single-nucleotide polymorphism (SNPs), with drug toxicity and efficacy. Phamacogenomics uses whole genome wide sequencing or target oriented sequencing to identify single gene interaction with drugs. It has been extensively studied in patients of cancer, tuberculosis, cardiac disorders, diabetes, asthma and HIV.

AIDS offers the greatest threat to humans of all infectious diseases in history. To its challenge, more than 25 FDA approved antiretroviral drugs are available for clinical use which target HIV reverse transcriptase, protease, or viral entry receptors. The lifelong administration of multiple drugs necessiates constant monitoring of drug efficacy. Though these medications significantly reduce AIDS-related mortality[1] but their efficacy is not only compromised by their toxicity, viral resistance, and nonadherence to treatment, but also by comorbidities lik hepatitis, diabetes, and cardiovascula disease.

The field of pharmacogenomics strive to understand relationship between human genetic variations and response to treatment.[2–6] The relevance of pharmacogenomics to HIV therapeutics spans basic science, patient care, and public health disciplines. Laboratory-based investigators use genomic techniques to study viral pathogenesis to explore new targets for therapeutic intervention.

The potential for human genetic research to identify novel therapeutic targets is highlighted by previous studies of CCR5. This cellular chemokine receptor is required for infectivity of many HIV strains.[7–9] Soon after its role in HIV replication was elucidated, individuals were identified who were highly resistant to HIV infection and lacked functional CCR5 as the result of a 32-bp deletion in the CCR5 gene but were otherwise healthy.[10–12] This experiment of nature suggested that CCR5 inhibitors could be effectiv antiretroviral agents, and several CCR5 inhibitors are now under clinical trials. Other cellular factors which restrict HIV replication are: mRNA-editing enzyme ‘apolipoprotein B’, a catalytic polypeptide ‘3G (APOBEC3G)[13] and tripartite motif ‘5a (TRIM5a)’.[14] The naturally occurring variants in these and associated genes which affect progression of HIV disease are potential intervention targets.

Progress in pharmacogenomics require access to DNA specimens from large, well-characterized patient population by genetic investigators. The Adult AIDS Clinical Trials Group (AACTG), funded by the National Institutes of Health, has created an important repository. Since 1986, the AACTG has enrolled 136,000 individuals into diverse prospective trials with well-defined entry criteria and on-study evaluations. To establish a usable DNA bank, a group of clinical researchers, genetic investigators, ethicists, statisticians, data managers, regulatory specialists, and community representatives worked in collaboration to develop AACTG Protocol A5128, which allows prospective study on stored DNA wher informed consent was obtained for other AACTG trials.[15] One challenge for the identification of genetic associations in cohort studies is to define control group with all relevant factors except the phenotype.[16]

Antiretroviral treatment is characterized by differential rates of adverse events and responses in seropositive individuals. Genetic variations between human beings are the major cause for this variablity. A number of associations of genetic variants with predisposition to drug adversities are well characterized, like hypersensitivity to abacavir. Although the drug is generally well tolerated, 5%–9% of Caucasians who receive abacavir experience hypersensitivity reactions proove life threatening unless intervened. Two research groups independently reported an association between major histocompatibility complex alleles and hypersensitivity to abacavir.[17,18] In patients exposed to abacavir in Perth, Australia, the presence of HLA-*015701, HLA-DR7, and HLADQ3 had a positive predictive value of 100% and a negative predictive value of 97% for hypersensitivity[18] An association between hypersensitivity to abacavir and HLA-*B5701 and HLA-DR7 was confirmed in patients in North America.[19] More-recent analyses have extended this association to include a polymorphism in Hsp70-Hom, a member of the heat shock protein family of chaperonins[19] HLA Class II allele DRB1*0101 has been associated with Nevirepine-associted hypersentivity.[20] HLA *013505 allele has been a strong predictor for neverpine-induced skin adverse drug reactions in Thai HIV patients.[21] In nevirapine induced rash HIV-1 positive infected individuals from Mumbai, India a highly significant association with HLA B35 an protection with HLA B7 is found.[22]

The nonnucleoside reverse-transcriptase inhibitor efavirenz is one of the most widely prescribed antiretroviral medications[23,24] but many recipients of efavirenz experience central nervous system side effects during the initial weeks of therapy.[24] Efavirenz is metabolized primarily by hepatic cytochrome P450 (CYP) 2B6[25] and a large amount of interindividual variability in the amount of CYP2B6 in the liver has been reported[26–29] as have functional differences between genetic variants.[28,30–32] Specimens from the AACTG Human DNA Repository and associated data from clinical trials were used to show that a CYP2B6 exon 4 polymorphism that occurs more frequently in blacks than in whites is associated with ~3-fold higher plasma concentrations of efavirenz (P.000)) and with increased central nervous system side effects (P p.036).[33] Differences in the frequency of this polymorphism in different populations may explain the lower clearance of efavirenz noted in blacks.[34–36] Recently the importance of human CYP3A pharmacogenetics with the discovery of the Null allelle CYP3A4*20 have contributed in predicting efficacy and/or toxicity in HIV patients.[37] The CYP3A4*1B polymorphism influences the pharmacokinetics of indinavir and to some extent its biochemical safety.[38]

Tumor necrosis factor (TNF)-α has been implicated in the pathogenesis of lipodystroph[39–41] and TNF-α expression varies according to race and ethnicity.[42] Two research groups have reported relationships between antiretroviral-associated lipodystrophy and a TNF-α promoter polymorphism that may affect gene expression. In 96 white patients in England, a TNF–α position 238 polymorphism was present only in subjects with lipodystrophy (P p .0)).[43,44] These findings support a role for TNF-α in the pathogenesis of lipoatrophy, this variant allele may simply be a marker for other genes with which it is linked, such as members of the major histocompatibility complex.[42]

Bilirubin is the primary product of heme metabolism. Its efficient elimination requires conjugation with glucuronic acid in a reaction catalyzed by hepatic UDPglucuronosyltransferase (UGT) 1A1. Approximately 5%–10% of individuals have decreased bilirubin-conjugating activity that is caused by a TA insertion into the UGT1A1 promoter (Gilbert syndrome).[45,46] The HIV protease inhibitors indinavir and atazanavir commonly cause unconjugated hyperbilirubinemia by competing with bilirubin for binding to UGT1A1. The HIV protease inhibitors are substrates for P-glycoprotein, the multidrug efflux pump encoded by MDR1[47] and a frequent MDR1 exon 26 polymorphism has been associated with altered P-glycoprotein expression.[48] P-glycoprotein in the intestine, liver, and kidney is predicted to decrease oral bioavailability of these drugs and enhance their elimination. P-glycoprotein is also present in CD4 T cells,[49] and its expression in the brain limits entry of protease inhibitors. Importantly, a provocative report noted an association between the MDR1 exon 26 polymorphism, increases in CD4 T cells in response to antiretroviral therapy, and plasma concentrations of efavirenz and nelfinavir.[50]

As pharmacogenomics moves from bench to bedside, most genotype-phenotype relationships will reflect the combined influences of multiple genes and polymorphisms. The growing number of identified genetic associations will increase the impetus to make human genetic testing a routine part of HIV clinical care. Prospective clinical trials will ultimately be needed to determine whether the use of human genetic testing to guide the administration of antiretroviral therapy results in an improved response to treatment. Because genetic variants are stable throughout one’s lifetime, genetic testing performed on a single occasion could potentially inform every subsequent treatment decision for a patient, and this makes such an approach to HIV clinical care even more attractive. Such approaches promise advent of “personalized medicine” in which drugs and drug combinations are optimized for each patients individual genetic makeup.
